# Geometry‐Driven Biomechanical Optimization of Double‐J Ureteral Stents

**DOI:** 10.1155/bmri/9681542

**Published:** 2026-11-15

**Authors:** Achmad Syaifudin, Mudhita Zikkrullah Ritonga, Lukman Hakim, Mohamad Taufiqurrakhman, Hemant Pandit

**Affiliations:** ^1^ Department of Medical Technology Institut Teknologi Sepuluh Nopember Surabaya Indonesia its.ac.id; ^2^ Center for Appropriate Technology Directorate of Research and Community Service (DRPM) Institut Teknologi Sepuluh Nopember Surabaya Indonesia its.ac.id; ^3^ Faculty of Veterinary Medicine Universitas Syiah Kuala Banda Aceh Aceh Indonesia unsyiah.ac.id; ^4^ Department of Urology Faculty of Medicine Universitas Airlangga Surabaya Indonesia unair.ac.id; ^5^ Leeds Institute of Rheumatic and Musculoskeletal Medicine (LIRMM) School of Medicine University of Leeds Leeds UK leeds.ac.uk

**Keywords:** biomechanics, double-J ureteral stent, geometric optimization, medical device design, stent migration

## Abstract

Ureteric stents are widely used to maintain ureteric patency; however, complications such as stent migration, ureteric irritation, and patient discomfort remain common. Although several geometric parameters of double‐J stents have been investigated in relation to urinary flow, stenosis, reflux, encrustation, stent length, and coil characteristics, the specific biomechanical role of terminal coil orientation relative to physiological loading remains insufficiently characterized. This study proposes a geometry‐based biomechanical framework that considers terminal coil‐plane orientation as a specific and mechanistically insufficiently characterized geometric variable in double‐J ureteral stent stability. The framework integrates biomechanical principles, engineering concepts, and clinical considerations to provide a simplified mechanistic interpretation of how coil geometry may interact with multidirectional physiological loading generated by ureteral peristalsis and intravesical pressure. Changes in coil‐plane orientation may alter the transmission and decomposition of physiological forces, potentially influencing axial loading, transverse forces, bending response, stent–wall contact, and resistance to longitudinal displacement. An alternative terminal coil‐plane orientation is proposed as a testable geometric modification that may alter these mechanical relationships without changing the overall dimensions or material composition of the device. The proposed relationships are conceptual and do not represent validated stress distributions, experimentally demonstrated retention benefits, or evidence that any particular coil orientation is superior. This framework provides a structured basis for future finite element analysis, computational fluid dynamics, and fluid–structure interaction studies, followed by experimental and clinical validation, to quantitatively evaluate coil orientation as a complementary geometric variable in double‐J ureteral stent design and development.

## 1. Introduction

Ureteric stents are widely used in urological practice to maintain urinary drainage and preserve ureteral patency in conditions, including ureteral obstruction, urinary stones, malignancy‐related compression, and following endourological procedures [1]. Among available devices, the double‐J stent remains one of the most widely used designs because its proximal and distal coils provide positional restraint and help reduce displacement after placement [2]. Despite their routine clinical use, stent‐related complications remain common, including migration, urinary irritation, infection, flank pain, and reduced patient comfort [3]. The continued occurrence of these complications suggests that current stent designs may not fully account for the complex mechanical conditions of the urinary tract [4, 5].

Innovation efforts to improve ureteral stents have involved both material‐ and geometry‐based approaches aimed at improving device performance and reducing stent‐related complications [6]. Material‐based innovations include advanced polymeric and biodegradable materials, metal stents, and various surface coatings, which have primarily been developed to reduce biofilm formation, encrustation, and patient discomfort [7]. In parallel, geometric and structural modifications, including lumen diameter, device flexibility, stent size, and side hole configuration, have been investigated to improve urinary drainage and overall stent performance [8]. Despite these advances, mechanical complications, such as stent migration and tissue irritation, continue to be reported in clinical practice, indicating that additional mechanical factors may contribute to the interaction between ureteral stents and the dynamic urinary tract environment [9].

Importantly, geometric design itself has not been neglected in ureteral stent research. Several computational and experimental studies have demonstrated that specific geometric parameters can influence urinary drainage under different anatomical and pathological conditions. Kim et al. [10] used computational fluid dynamics to compare different side‐hole arrangements and reported a higher total urine flow rate for stents with side holes distributed along the entire shaft (16.5 mL/h) than for stents with side holes restricted to the proximal and distal shaft segments (12.7 mL/h) under ureteral stenosis. Similarly, in curved anatomy‐based ureteral models with stenosis, the presence and number of side holes influenced urinary drainage, with stents containing side holes producing higher luminal and total flow rates, particularly under severe stenosis, whereas the angular position of the side holes did not significantly affect the flow rate [11]. In a broader computational fluid dynamics analysis comprising 64 simulations combining different ureteral diameters, degrees of stenosis, and double‐J stent sizes, increasing stent size was generally associated with decreased total urine flow under the modeled conditions [12]. Furthermore, Kim et al. [13] combined computational fluid dynamics with an in vitro silicone ureter model to evaluate 5–8 Fr double‐J stents and found that the 5 Fr stent produced the highest total flow rate, whereas urinary flow decreased with increasing stent size. These findings demonstrate that geometric parameters such as side‐hole distribution, stent size, and lumen‐related configuration can influence the hydrodynamic performance of ureteral stents.

Geometric configuration has also been investigated in relation to encrustation, reflux, and alternative stent designs. In an artificial urine model, encrustation accumulated preferentially in the distal region of double‐J stents and around the side holes, with the encrustation area increasing over time and progressively obstructing the side holes [14]. Previous computational fluid dynamics modeling of the urinary tract with double‐J stents demonstrated that bladder pressure and ureterovesical junction dynamics were associated with reflux behavior, highlighting the importance of considering distal coil–ureterovesical junction interactions when evaluating alternative coil configurations [15]. Modified double‐J stents with additional side holes increased urine flow in nonstenotic models but showed no significant advantage over conventional stents under stenotic conditions [16]. More recent in vitro and computational fluid dynamics analyses reported that modified double‐J stents provided the highest flow performance, whereas single‐J stents exhibited the lowest encrustation under ureteral stenosis [17]. Taken together, these studies demonstrate that stent geometry and configuration can influence urinary flow, reflux, and encrustation under different physiological and pathological conditions.

However, these investigations have primarily focused on hydrodynamic performance, drainage, reflux, and encrustation, whereas the biomechanical consequences of terminal coil‐plane orientation relative to physiological loading remain insufficiently characterized. Numerical analyses using three anatomically based ureteral models demonstrated that ureteral geometry influenced urine flow rate and flow patterns, whereas the contribution of the double‐J stent side holes to flow diversion varied according to ureteral anatomy [18]. Such findings highlight the importance of anatomical and geometric factors in determining stent performance but also indicate that hydrodynamic behavior alone does not fully capture the mechanical response of ureteral stents. From a biomechanical perspective, the relationship between terminal coil‐plane orientation and physiological force vectors represents a more specific geometric question that has not yet been quantitatively established.

The clinical relevance of stent orientation has recently been demonstrated by Bayar et al. [19], who conducted a prospective randomized controlled study comparing conventional and reverse placement of double‐J ureteral stents in 120 patients undergoing ureteroscopic stone treatment. Reverse placement was associated with lower ureteral stent symptom scores, reduced analgesic use, and less frequent midline crossing of the stent, although subgroup findings varied according to the orientation of the upper and lower stent ends [19]. These findings provide clinical evidence that stent placement orientation can influence patient‐reported symptoms and stent positioning. However, reverse clinical placement represents a positional configuration of the stent and should be distinguished from terminal coil‐plane orientation. Therefore, the clinical study provides evidence for orientation‐dependent differences in stent behavior but does not establish the biomechanical mechanisms responsible for these differences. The relationships among coil‐plane orientation, physiological force vectors, force transmission, stress distribution, stent–tissue contact, urinary flow, and resistance to displacement remain insufficiently characterized and have not yet been quantitatively established.

Geometry is a fundamental determinant of the mechanical performance of biomedical devices. The structural configuration of an implant, together with its material properties, can influence mechanical stability, stress distribution, deformation, and interaction with surrounding biological tissues [20, 21]. Even relatively small changes in implant geometry may alter its mechanical behavior and consequently affect its long‐term clinical performance. The ureter is a dynamic muscular organ that transports urine through rhythmic peristaltic contractions [22]. These contractions generate time‐varying mechanical forces along the ureteral lumen, whereas fluctuations in intravesical bladder pressure impose additional loading on the distal segment of the stent [23]. The interaction between physiological loading and stent geometry therefore creates a complex biomechanical environment in which terminal coil‐plane orientation may influence force transmission, deformation, and stent–tissue interaction. Because the distal coil resides within the bladder near the ureterovesical junction, its orientation may also influence local stent–tissue contact and urinary flow conditions under changing intravesical pressure. The ureterovesical junction functions as a pressure‐regulating barrier between the bladder and upper urinary tract, and its pressure relationship with the bladder is relevant to vesicoureteral reflux [24]. Previous computational studies have further demonstrated that bladder pressure and ureterovesical junction dynamics can influence reflux behavior in urinary tracts containing double‐J stents [15]. These observations indicate that the distal coil operates within a pressure‐sensitive anatomical environment in which coil geometry may interact with both local tissue contact and urinary flow [25, 26].

Several geometric variables should be distinguished when describing the coil configuration. In this framework, coil orientation specifically refers to the spatial orientation of the coiled segment relative to the stent shaft and the predominant direction of physiological loading. Reverse placement refers to the end‐to‐end reversal of a double‐J stent during clinical insertion, whereas winding handedness describes the directional sense of coil winding. Other geometric variables include coil pitch and turn count, which describe the spacing and number of coil turns, respectively. These variables represent distinct aspects of stent geometry and should not be used interchangeably. Thus, the present framework focuses specifically on terminal coil‐plane orientation rather than winding handedness, pitch, turn count, or reverse clinical placement. Coiled segments provide an important geometric contribution to positional retention and resistance to migration, whereas previous studies have primarily examined factors such as coil turns, stent stiffness, and retention‐related mechanical properties [27]. Changes in coil‐plane orientation may modify the direction and distribution of loads transmitted through the coiled segment and may consequently influence structural deformation, local stress, stent–wall contact, and frictional resistance [28, 29]. Such geometric modifications may alter the mechanical response and resistance to displacement without requiring changes in the material composition or overall dimensions of the stent [30] (Figure 1).

**Figure 1:**
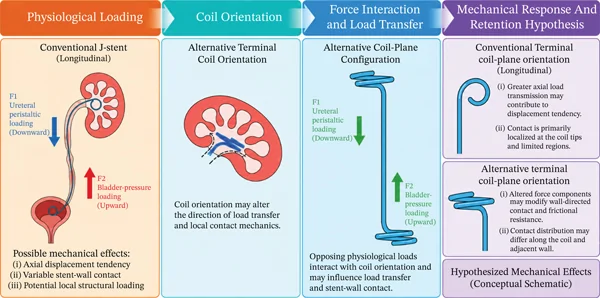
Conceptual biomechanical framework illustrating how physiological loading, terminal coil‐plane orientation, and load transfer may influence stent–wall contact and resistance to displacement in double‐J ureteral stents. The schematic compares a conventional terminal coil‐plane orientation with an alternative terminal coil‐plane orientation as a hypothesis‐generating geometric concept. The illustration is qualitative and does not represent finite element analysis, experimental measurements, or validated mechanical outcomes. The proposed relationships among force transmission, structural response, stent–tissue interaction, and resistance to displacement require quantitative evaluation.

This study presents a hypothesis‐driven biomechanical perspective on the design of double‐J ureteral stents. Rather than suggesting that ureteral stent geometry has been neglected, the present framework focuses on a more specific and insufficiently characterized question: Whether the orientation of the terminal coil relative to physiological loading may influence stent stability. This study proposes that an alternative terminal coil‐plane orientation may modify the manner in which physiological forces are resolved and transmitted through the coiled segment, potentially affecting local deformation, bending response, stent–wall contact, frictional resistance, and resistance to longitudinal displacement. This hypothesis does not assume that a particular coil orientation provides superior retention; rather, it identifies coil‐plane orientation as a testable geometric variable that can be evaluated without altering the overall stent dimensions or material composition. The aim is to develop a simplified conceptual framework linking coil geometry, physiological loading, structural response, and stent–tissue interaction, while complementing existing research on antimigration geometry, fluid‐mechanical optimization, and stent structural mechanics. The proposed relationships provide a basis for future evaluation using computational fluid dynamics, finite element analysis, and fluid–structure interaction, followed by experimental and clinical validation.

## 2. Current Design of Ureteral Stents and Limitations

Ureteric stent development has progressed through both material‐based and geometry‐based strategies aimed at improving urinary drainage, positional stability, biocompatibility, and patient comfort [6–8, 31–33]. Material and surface modifications have included advanced polymeric and biodegradable materials, metal stents, and various surface coatings, with particular emphasis on reducing biofilm formation, encrustation, infection, and stent‐related discomfort [7, 34]. In parallel, geometric and structural modifications have addressed stent diameter, lumen dimensions, flexibility, side‐hole configuration, coil characteristics, stent length, and implantation position [8, 27, 28, 35–38]. Thus, geometric design itself cannot be considered neglected in ureteral stent research. Rather, different geometric features have been investigated for specific functional objectives, whereas some aspects of their biomechanical interaction with the dynamic urinary tract environment remain insufficiently characterized.

Antimigration geometry is an important component of ureteral stent development. Earlier designs incorporated features such as distal flanges and barbs to resist displacement, followed by double‐pigtail and double‐J configurations in which the curled ends provide positional restraint within the renal pelvis and bladder [8, 31, 32]. Other modifications, including tapered distal ends and alternative terminal configurations, have also been investigated in relation to drainage, patient comfort, and positional stability [36]. These developments demonstrate that stent‐end geometry has long been clinically relevant. However, most geometric modifications have focused on anchoring shape, overall configuration, drainage, or patient‐related outcomes rather than on the directional relationship between terminal coil geometry and physiological loading.

Computational and experimental studies have further demonstrated that geometric parameters influence urinary flow and drainage. Numerical models of stented ureters have examined the effects of ureteral geometry, local obstruction, stent diameter, lumen dimensions, and side‐hole configuration on urinary flow [25, 36]. In particular, the number and position of side holes have been shown to modify intraluminal and extraluminal flow patterns, particularly in curved or obstructed ureteral models [36]. Other studies have examined side‐hole diameter, lumen dimensions, local wall shear stress, and additional side‐hole placement [39, 40]. These findings provide an important basis for the geometry‐driven optimization of urinary drainage. However, their primary emphasis has been on hydrodynamic performance rather than the mechanical contribution of terminal coil geometry to anchoring, contact mechanics, or resistance to migration.

A separate body of research has addressed the structural and mechanical behavior of stents. Simplified mechanical models have been used to relate stent geometry to stiffness, deformation, and load‐bearing behavior [38], whereas theoretical and numerical analyses of other stent systems have demonstrated that structural geometry can influence deformation and mechanical responses under applied loading [28]. Although these studies were not specifically focused on the terminal coils of double‐J ureteral stents, they provide a general mechanical basis for considering geometry as a determinant of load transmission and structural response. Within the ureteral context, experimental characterization has shown that stent behavior under external compression depends on the structural and material properties [41], whereas fluid–structure modeling has demonstrated the feasibility of computationally representing the interactions between a stent, ureter, and urinary flow [36].

Migration resistance provides another relevant mechanical perspective. Computational studies of other stent systems have demonstrated that stent geometry, contact conditions, and friction can influence resistance to displacement [29]. Because these findings were obtained from esophageal rather than ureteral stents, they should not be quantitatively extrapolated to double‐J ureteral stents. Nevertheless, they provide a useful mechanical precedent for considering stent migration as a balance between displacement‐driving forces and contact‐related resistance. In ureteral stents, clinical and experimental studies have examined migration, stent positioning, coil configuration, and reverse placement [3, 19, 27, 32]. These studies establish the clinical and mechanical relevance of terminal geometry and positioning but do not directly establish how terminal coil‐plane orientation modifies local force transmission, stress distribution, or stent–tissue contact.

Taken together, the available literature demonstrates that ureteral stent geometry has been investigated across multiple domains, including antimigration features, terminal‐end configuration, stent diameter and length, lumen geometry, side‐hole number and placement, implantation position, urinary flow performance, compression response, and stent positioning [19, 27, 35, 39, 40]. Therefore, the remaining gap is not the absence of geometric research but the limited characterization of a specific geometric–mechanical relationship: the orientation of the terminal coil plane relative to physiological loading and its potential influence on force transmission, structural response, stent–tissue contact, and resistance to longitudinal displacement in double‐J ureteral stents.

Accordingly, terminal coil‐plane orientation may be considered an additional geometric variable operating alongside established factors, such as coil curvature, turn count, pitch, stent diameter, material stiffness, surface properties, anatomical constraints, and physiological loading. The proposed perspective does not assume that any particular coil orientation provides superior retention. Rather, it identifies coil‐plane orientation as a testable biomechanical variable whose effects can be quantitatively investigated using finite element analysis, computational fluid dynamics, and fluid–structure interaction, followed by anatomically representative experimental and ultimately clinical evaluation. Such an approach may complement existing material‐, hydrodynamic‐, and geometry‐based strategies by providing a more specific framework for understanding how terminal coil geometry interacts with the mechanical environment of the urinary tract.

The mechanical environment of the urinary tract further complicates this relationship. The ureter operates within a complex and dynamic mechanical environment in which peristaltic contractions and variations in intravesical pressure may generate longitudinal and retrograde loading conditions, respectively [22, 24, 40]. These forces interact with urine flow, luminal geometry, ureteral curvature, tissue contact, and local obstruction and may contribute to multidirectional loading on intraluminal devices [31]. Computational models have captured several aspects of this environment, particularly urinary flow, obstruction, reflux, and side‐hole performance. In particular, a computational fluid dynamics analysis has demonstrated that vesicoureteral reflux in urinary tracts containing double‐J stents can vary with bladder pressure, ureteral stenosis, and urinary tract dynamics [15]. However, the terminal coil has generally been considered primarily as a geometric retention feature rather than as a structural component, whose spatial orientation may modify the transmission and distribution of physiological loads. This distinction is particularly relevant because the direction of loading relative to the coil plane may affect axial force transmission, bending response, wall‐directed contact, and frictional resistance (Figure 2).

**Figure 2:**
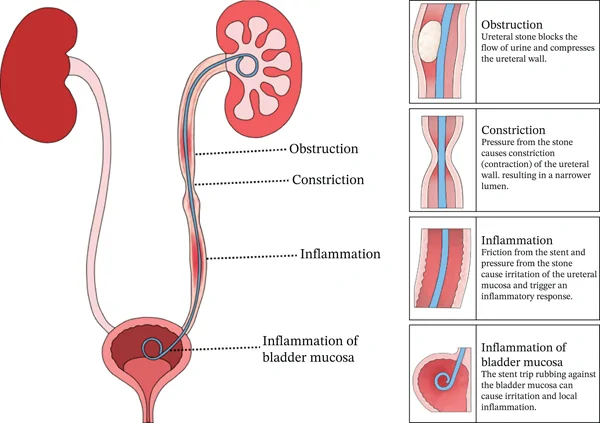
Biomechanical environment and physiological force interactions surrounding double‐J ureteral stent. The illustration depicts the anatomical placement of a double‐J stent within the urinary tract and representative physiological loading conditions, including ureteral peristalsis, intravesical pressure, luminal compression, and multidirectional force interaction. These dynamic conditions may influence force transmission, stent deformation, stent–ureteral wall contact, and resistance to longitudinal displacement of the stent. The illustration is qualitative and provides anatomical and biomechanical context for the proposed framework, rather than representing a quantitative mechanical or computational analysis.

## 3. Biomechanical Environment of the Ureter

The ureter is a dynamic organ, and a stent placed within it is continuously exposed to changing mechanical conditions. Ureteral peristalsis generates loading that acts predominantly along the ureteral axis, whereas changes in bladder pressure may produce loading from the distal side of the stent [23, 42].

These forces do not necessarily act independently or remain constant over time. Instead, their combined action creates a variable loading environment that may affect stent displacement, deformation, and interaction with the ureteral wall.

The proximal and distal coils are particularly important because they provide the main geometric restraint against stent displacement. Their ability to maintain stent position may depend not only on coil size and stiffness, but also on their orientation relative to the applied loading. Previous mechanical studies have shown that stent geometry can influence deformation, structural response, and resistance to external loading [28, 38]. Although these studies were not specifically designed to evaluate alternative terminal coil‐plane orientations in ureteral stents, they support the broader mechanical principle that geometric configuration can influence load transmission through a stent structure.

A simplified force representation can be used to clarify this relationship. The physiological loading acting on the stent may be represented as the vector sum of the principal contributions:

(1) Fphys=Fperi+Fblad+Fother,

where *F*_*p* *e* *r* *i*_ represents loading associated with ureteral peristalsis, *F*_*b* *l* *a* *d*_ represents loading associated with bladder pressure, and *F*_*o* *t* *h* *e* *r*_ accounts for additional contributions such as local wall contact and anatomical constraint. This expression is not intended to reproduce the complete in vivo loading condition. Rather, it provides a simplified representation of the principal forces considered in the present framework.

The orientation of the coil determines how the resultant physiological load is resolved relative to the local coil direction. If *θ* is defined as the angle between the resultant force and the local tangent of the coil, the magnitude of the load can be decomposed into parallel and perpendicular components:
(2)

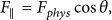

(3) F⊥=Fphyssinθ.

The parallel component, *F*_‖ _, represents the portion of the load transmitted along the local coil direction. When this direction is aligned with a possible displacement pathway, this component may contribute to axial stent movement. The perpendicular component, *F*_⊥_, acts across the local coil direction and may contribute to coil deformation and wall‐directed contact. Consequently, changing the coil orientation changes the relative magnitudes of these two components even when the magnitude of the external load remains unchanged.

Coil orientation may also influence the bending response of the stent. In a simplified mechanical representation, the bending moment generated by the applied force may be expressed as:

(4) M=rFphyssinθ,

where *r* represents the effective moment arm. This relationship suggests that a change in coil orientation may alter the balance between axial loading and bending. At a first‐order level, the resulting structural stress may be represented as a combination of axial and bending contributions:
(5)

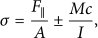


where *A* is the effective cross‐sectional area, *I* is the second moment of area, and *c*is the distance from the neutral axis. This expression is included only to illustrate the mechanical connection between force direction, coil geometry, and structural loading. It should not be interpreted as a prediction of the actual stress field within a ureteral stent. Local stress patterns would depend on three‐dimensional geometry, material behavior, contact conditions, and anatomical constraints and would require computational or experimental analysis. This limitation is already consistent with the cautionary statement later in the section that the present framework does not demonstrate lower stress or improved retention.

Contact between the coil and surrounding tissue provides another possible contribution to stent retention. A component of the applied load directed toward the tissue may generate a normal contact force, *N*. Using a simplified Coulomb‐type friction model, the available frictional resistance may be represented as follows:

(6) Ffriction≤μN,

where *μ* represents the effective coefficient of friction at the stent–tissue interface. Previous work on stent migration has shown that changes in stent geometry can affect contact interaction and resistance to displacement [29]. Because that study involved an esophageal stent, its findings cannot be transferred quantitatively to the ureter. Nevertheless, the underlying mechanical principle remains relevant: geometry can influence contact force and, consequently, resistance to migration.

From this perspective, stent migration can be viewed as a balance between displacement‐driving and resisting forces. In a simplified form, the resisting force may be written as follows:

(7) Fresist=Fanchor+Ffriction,

where *F*_*a* *n* *c* *h* *o* *r*_ represents the mechanical resistance associated with coil deformation and geometric engagement. Migration would become more likely when the displacement‐driving component of the applied load exceeds the available resisting force.

This simplified representation helps explain why coil orientation may be relevant to stent stability. Two coil configurations exposed to the same physiological loading may not transmit that loading in the same way. A change in orientation alters the angle *θ*, which in turn changes the balance among parallel loading, perpendicular loading, bending, and wall contact. On this basis, an alternative terminal coil‐plane orientation is hypothesized to modify the proportion of loading acting along a potential migration direction and the contribution of deformation and contact‐related resistance.

Importantly, this interpretation does not demonstrate that the proposed orientation produces lower stress or better retention. It provides a mechanical basis for a testable hypothesis. Quantitative differences between conventional and alternative coil configurations would need to be evaluated using finite‐element analysis, benchtop experiments, or other appropriate validation methods (Figure 3).

**Figure 3:**
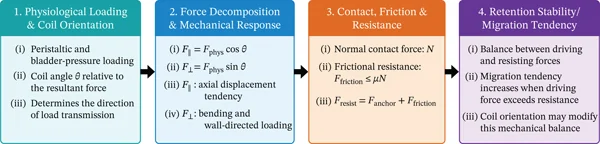
Simplified mechanistic framework linking physiological loading, coil orientation, and stent retention. Physiological loading is resolved relative to the local coil orientation into parallel and perpendicular components. These components may influence axial load transmission, bending, contact interaction, and frictional resistance, thereby affecting the balance between displacement‐driving and resisting forces. The relationships shown are conceptual and are not derived from finite‐element analysis or experimental stress measurements.

## 4. Coil Orientation and Force Interaction

The simplified mechanical framework described above provides a basis for examining the coil orientation as a geometric factor in stent stability. Rather than assuming that one orientation is mechanically superior, the framework considers how changes in the coil direction may alter the transmission of physiological loading through the stent. In the conventional double‐J configuration, the proximal and distal coils primarily provide geometric restraint against displacement; however, their mechanical response depends on the coil curvature, orientation, and direction of the applied load.

For a given physiological load, the local orientation of the coil determines the angle *θ* between the resultant force and the local coil direction. As described in Section 3, this angle affects the relative magnitudes of the parallel and perpendicular force components. A larger parallel component, *F*_‖ _, may contribute to load transmission along a potential displacement pathway, whereas a larger perpendicular component, *F*_⊥_, may contribute to coil deformation and wall‐directed contact. Changing coil orientation may therefore alter the balance between displacement‐driving and resisting mechanical effects.

In the conventional double‐J configuration, the proximal and distal coils primarily provide geometric restraint against displacement. Their mechanical response, however, depends on the local curvature, coil orientation, and direction of the applied load. If a substantial proportion of physiological loading is transmitted along the stent axis, the parallel force component may contribute to axial displacement. In contrast, an alternative terminal coil‐plane orientation may alter the local relationship between the applied force and the coil direction, thereby changing the relative contributions of axial loading, bending, and tissue contact.

This change in load transmission may also influence contact mechanics at the stent–tissue interface. An increase in wall‐directed loading may increase the normal contact force, *N*, which may in turn increase the available frictional resistance according to the simplified relation, as indicated in Equation (6). In this framework, the possible mechanical effect of an alternative coil orientation does not arise simply from changing the shape of the stent, but from changing how external loading is transferred into parallel loading, bending, deformation, and contact‐related resistance.

Coil orientation may also affect the distribution of the structural loading within the stent. Changes in the force direction and effective moment arm may modify the relative contributions of axial and bending stresses. However, the present framework does not establish whether an alternative coil‐plane orientation produces a lower maximum stress or a more uniform stress distribution than the conventional configuration. Such differences cannot be determined from simplified force relationships alone and would require finite element analysis or experimental measurements.

The proposed mechanical relationships may have different implications for the renal and distal coils because these components interact with different anatomical structures. Under conditions of reduced or absent ureteral peristalsis, variations in urinary tract pressure may become relatively more important in determining stent position and tissue contact. In this context, an alternative renal coil‐plane orientation could potentially alter the extent and distribution of the contact with the renal pelvis. Increased or localized contact may contribute to mechanical irritation or patient discomfort; although this proposed effect remains hypothetical and requires quantitative evaluation [43, 44]. Similarly, the orientation and position of the distal coil may influence its interaction with the ureterovesical junction under varying intravesical pressures, with potential implications for local contact mechanics, urinary drainage and vesicoureteral reflux. These relationships remain hypotheses rather than demonstrated effects.

Accordingly, the proposed alternative coil‐plane orientation should be regarded as a testable design hypothesis rather than a demonstrated improvement. Its potential mechanical effects may arise from changes in the transmission of physiological loading into axial loading, bending, deformation, and contact‐related resistance. Whether these changes translate into measurable differences in resistance to longitudinal displacement will depend on additional factors, including coil curvature, material stiffness, surface properties, frictional conditions, anatomical constraints, and the magnitude and direction of physiological loading.

Therefore, geometry‐based modification may complement, rather than replace, existing material‐ and surface‐based approaches to ureteral stent development. The practical significance of coil orientation will ultimately depend on whether the proposed changes in load transmission, structural response, and contact mechanics can be demonstrated under physiologically relevant computational, benchtop, in vivo, and clinical conditions.

## 5. Design Propositions and Implications

The framework proposed in this study treats terminal coil‐plane orientation as a distinct geometric variable that may influence the mechanical and hydrodynamic behavior of double‐J ureteral stents. At this stage, however, the framework should not be interpreted as evidence that an alternative coil‐plane orientation performs better than the conventional configuration. Its purpose is to identify specific biomechanical relationships that can be examined experimentally and computationally while keeping other major design parameters, such as material properties and overall stent dimensions, comparable.

From a mechanical point of view, the response of the terminal coil is likely to depend not only on the magnitude of physiological loading but also on the direction in which that load acts relative to the local coil plane. A resultant force acting on the terminal segment can be considered in terms of components parallel and perpendicular to the coil plane. Changing the orientation of the coil may therefore change the relative contribution of these components. In turn, this could influence axial force transmission, wall‐directed loading, bending behavior, local deformation, stent–tissue contact, and frictional resistance.

The anatomical setting is also important. The proximal and distal coils are exposed to different mechanical environments. The proximal coil lies within the renal pelvis and is affected by ureteral peristalsis, upper urinary tract pressure, anatomical confinement, and contact with the renal pelvis. The distal coil, by contrast, is located in the bladder near the ureterovesical junction, where intravesical pressure and local anatomical interaction may play a greater role. For this reason, changes in terminal coil‐plane orientation may not produce the same mechanical response at both ends of the stent.

Figure 4 summarizes the anatomical and mechanical basis of the three propositions presented below. It illustrates the relationship between physiological loading, terminal coil‐plane orientation, force decomposition, and local stent–tissue interaction.

**Proposition 1.**

Terminal coil‐plane orientation may alter force transmission and resistance to longitudinal displacement.

The orientation of the terminal coil relative to physiological loading may influence how force is transferred through the stent. When the coil plane is changed, the relative axial and transverse components of the applied load may also change. This could affect the amount of force transmitted along the stent shaft and the amount redirected toward the surrounding anatomical structures.

Such a change may also influence longitudinal displacement. For example, a larger wall‐directed component could increase local contact and modify frictional resistance, whereas a larger axial component could contribute more directly to displacement along the stent axis. These relationships should be treated as hypotheses rather than established mechanical effects because they are likely to depend on anatomical geometry, surface contact, friction, material behavior, and the magnitude and direction of physiological loading.

A direct comparison between conventional and alternative terminal coil‐plane configurations would allow this proposition to be tested quantitatively under otherwise matched conditions. Relevant outcomes include axial and transverse force components, force transmission along the stent shaft, stent–tissue contact force, frictional resistance, longitudinal displacement, and the force required to initiate or maintain stent movement.

**Proposition 2.**

Terminal coil‐plane orientation may modify structural response and local contact mechanics.

Because the terminal coil is a curved structural element, its response to loading may vary with the direction of the applied force. A change in coil‐plane orientation may therefore influence bending, deformation, and the way mechanical stress is distributed within the coiled segment.

Different orientations may produce different deformation patterns even when the stent material and overall geometry remain unchanged. These changes may also affect where and how the coil contacts the surrounding tissue. The effect may differ between the renal pelvis and the bladder because the two regions provide different anatomical constraints and pressure environments.

This relationship could be examined using finite element analysis or fluid–structure interaction modeling. Useful quantitative measures would include stent deformation, bending response, stress distribution, contact area, contact force, and local tissue stress or strain. To isolate the effect of terminal coil‐plane orientation, these comparisons should use matched material properties, geometric dimensions, and physiological loading conditions.

**Proposition 3.**

Terminal coil‐plane orientation may influence pressure‐dependent stent–tissue interaction and local urinary flow.

The effect of terminal coil‐plane orientation may become more apparent when physiological pressure conditions change. At the proximal end, the mechanical environment of the renal coil may differ when ureteral peristalsis is reduced or absent. Under these circumstances, pressure changes within the upper urinary tract and contact with the renal pelvis may become more important contributors to stent behavior.

An alternative coil‐plane orientation could change the extent or distribution of contact between the renal coil and the renal pelvis. This may affect deformation, contact force, and the local flow environment. At the same time, greater contact should not automatically be considered beneficial. It may also contribute to irritation, discomfort, low‐flow regions, biofilm formation, or encrustation. These possibilities need to be examined rather than assumed.

At the distal end, intravesical pressure may affect the position and deformation of the coil near the ureterovesical junction. Depending on its orientation, the distal coil may interact differently with the bladder wall and the ureterovesical junction. Such changes could influence the effective luminal area, urinary drainage, and reflux‐related flow.

This proposition can therefore be evaluated under different levels of ureteral peristaltic activity and under varying upper urinary tract and bladder pressures. Computational fluid dynamics could be used to compare urinary flow rate, pressure distribution, pressure drop, velocity distribution, low‐flow or stagnation regions, wall shear stress, reflux‐related flow, and flow through the side holes. Fluid–structure interaction modeling could further evaluate contact area and force at the renal pelvis and ureterovesical junction, stent and ureteral deformation, local tissue stress or strain, effective luminal area, and pressure‐dependent mechanical loading.

**Figure 4:**
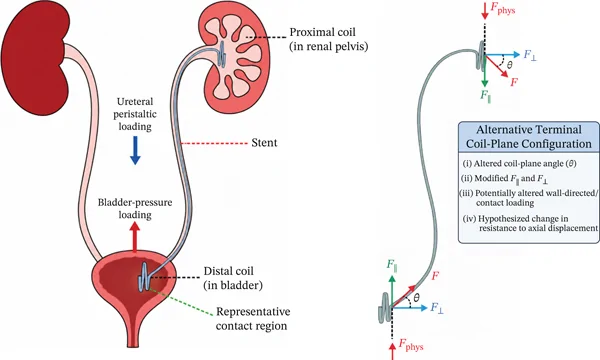
Conceptual anatomical and free‐body representation of an alternative terminal coil‐plane configuration in a double‐J ureteral stent. The anatomical schematic provides the physiological context of ureteral peristaltic and bladder‐pressure loading. The free‐body diagrams illustrate how a resultant local force (*F*) may be resolved into components parallel (*F*_‖ _) and perpendicular (*F*_⊥_) to the local coil plane. A change in the local coil‐plane angle (*θ*) may alter the balance between axial force transmission and wall‐directed contact loading. This illustration is intended as a conceptual mechanical representation and does not show measured stress distributions, finite‐element results, or demonstrated improvement in stent retention.

The three propositions address different aspects of the same biomechanical question. Proposition 1 focuses on force transmission and longitudinal displacement; Proposition 2 addresses deformation and contact mechanics; and Proposition 3 considers pressure‐dependent anatomical interaction and urinary flow.

These propositions should not be interpreted as evidence that the alternative orientation is superior to the conventional design. Rather, they define measurable relationships that can be tested in future studies. A staged approach combining finite element analysis, computational fluid dynamics, fluid–structure interaction modeling, anatomically representative in vitro testing, and later in vivo and clinical evaluation would be needed before drawing conclusions about migration resistance, urinary drainage, patient comfort, safety, or clinical benefit.

## 6. Limitations and Future Directions

This study presents a conceptual biomechanical framework and therefore does not provide experimental, computational, or clinical validation of the proposed terminal coil‐plane configurations. No finite element simulations or quantitative stress analyses were performed. Accordingly, the relationships proposed between coil‐plane orientation, force transmission, structural response, contact mechanics, and stent stability should be regarded as testable hypotheses rather than confirmed mechanical effects. The simplified force and stress representations used in this framework are intended to illustrate possible mechanical relationships and should not be interpreted as predictions of actual in vivo stress distributions, contact forces, or retention forces.

The framework also simplifies the ureteral environment by emphasizing ureteral peristalsis and intravesical pressure as the principal sources of physiological loads. In clinical conditions, stent behavior is influenced by patient‐specific anatomy, ureteral curvature, pathological narrowing, tissue compliance, urine flow, bladder dynamics, and variations in stent placement. These factors may modify the magnitude and direction of the applied loads and therefore limit the direct generalization of the proposed relationships to individual patients. In addition, coil orientation cannot be considered independently of other device characteristics. Material stiffness, coil curvature, stent diameter, surface properties, frictional conditions, lumen geometry, and local stent–tissue contact may interact with coil orientation and modify the overall mechanical response. These coupled effects were not quantitatively evaluated in the present framework and should be incorporated into future computational and experimental studies.

Future investigations should therefore proceed using a staged and complementary computational, experimental, and clinical validation strategy. Finite element analysis should first be used to compare conventional and alternative coil‐plane configurations under matched geometric and material conditions and physiologically relevant loading. Such models should incorporate three‐dimensional coil geometry, realistic material properties, stent–tissue contact conditions, frictional interactions, and representative loading at the renal pelvis, ureter, and ureterovesical junction. Key structural outcomes should include stent and ureteral deformation, axial and bending stresses, contact force and contact area, local tissue stress or strain, and resistance to longitudinal displacement. This approach would allow the specific contribution of the coil‐plane orientation to be examined while controlling other major design variables.

Computational fluid dynamics simulations should be performed in parallel to evaluate the potential hydrodynamic consequences of coil orientation. Conventional and alternative coil‐plane configurations should be compared under matched stent length and diameter, material properties, side‐hole number and size, and anatomical boundary conditions. Simulations should incorporate physiologically relevant variations in intravesical and upper urinary tract pressure, together with normal, reduced, and absent ureteral peristaltic activity. Relevant hydrodynamic outcomes should include the total urine flow rate, pressure drop, local velocity distribution, low‐flow or stagnation regions, wall shear stress, reflux flow or reflux tendency, and flow through the stent side holes. This computational design would allow the structural and hydrodynamic consequences of coil orientation to be evaluated without confounding them with unrelated changes in stent geometry or material composition.

Fluid–structure interaction analyses should be used to integrate fluid and structural responses under coupled physiological conditions. These models should evaluate stent and ureteral deformation, stent–tissue contact area and force, local tissue stress or strain, effective luminal area, and mechanical loading at the renal pelvis and ureterovesical junction. Particular attention should be paid to pressure‐dependent changes in the distal coil–ureterovesical junction interaction and renal coil–renal pelvis contact. Previous fluid–structure interaction modeling of a stented ureter demonstrated that fluid–structure coupling can be incorporated using nonlinear constitutive models for the ureter, although the influence of fluid–structure interaction on urine flow was small under the simplified pressure conditions examined in that study [36]. Accordingly, fluid–structure interaction may be particularly informative for evaluating deformation and contact mechanics, whereas computational fluid dynamics can provide the primary assessment of hydrodynamic performance.

Computational predictions should be tested using anatomically representative in vitro urinary tract models. Existing urinary‐stent evaluation frameworks support progressive in silico, in vitro, and in vivo approaches for linking device design with clinically relevant performance [35, 36]. The same matched conventional and alternative coil‐plane configurations evaluated computationally should be tested under controlled physiological conditions to enable direct comparison between predicted and experimentally measured outcomes. Relevant experimental endpoints should include urinary flow rate, drainage performance, pressure changes across the stented segment, stent displacement or resistance to displacement, coil deformation, and evidence of flow restriction or obstruction. Where appropriate, imaging‐based techniques may also be used to characterize local flow patterns and stent–tissue interactions around the renal and distal coils. The experimental models should also allow controlled variation of relevant parameters, such as friction, stiffness, anatomical constraints, intravesical pressure, and peristaltic activity.

Longer term in vitro studies should also investigate whether orientation‐dependent changes in local flow or stent–tissue contact are associated with biofilm formation and encrustation. Quantitative assessment of the encrustation burden, surface coverage, distribution around coil segments and side holes, and changes in flow performance over time could help determine whether an alternative coil‐plane configuration introduces additional risks associated with altered local flow or tissue contact. These studies would extend the proposed framework beyond short‐term mechanical stability and address the potential long‐term consequences of modifying the terminal coil geometry.

Finally, in vivo and clinical studies are required to determine whether the mechanical and hydrodynamic differences identified through computational and in vitro testing remain relevant under the greater anatomical and physiological complexity of the living urinary tract. Relevant clinical outcomes may include stent migration, ureteral or bladder irritation, urinary obstruction, reflux‐related effects, patient‐reported symptoms, and overall device performance. Patient‐specific anatomical and physiological variability should be incorporated into these studies to determine whether the effects of coil orientation are consistent across different clinical conditions.

Taken together, this staged finite element analysis–computational fluid dynamics–fluid–structure interaction–in vitro–in vivo/clinical workflow would provide a progressive pathway for evaluating the proposed biomechanical relationships. The primary objective should not be to assume that an alternative coil‐plane orientation is superior to the conventional configuration but to determine quantitatively whether changing coil orientation produces measurable differences in force transmission, structural response, stent–tissue interaction, urinary drainage, reflux behavior, and long‐term biofilm or encrustation characteristics. Only after these relationships have been demonstrated under physiologically relevant conditions can coil‐plane orientation be appropriately evaluated as a complementary geometric variable in future ureteral stent development.

## 7. Conclusion

This study proposes a geometry‐based biomechanical perspective on double‐J ureteral stent design, with particular attention to terminal coil‐plane orientation as a mechanistically insufficiently characterized geometric variable. Recent clinical evidence has shown that reverse stent placement may influence stent‐related symptoms and positioning; however, the biomechanical mechanisms underlying these observations remain poorly characterized. The present framework extends this clinical observation by considering the coil‐plane orientation as a testable geometric variable that may alter the interaction between the physiological force vectors and stent structure, with potential consequences for force transmission, bending and deformation, stent–tissue contact, urinary flow, and resistance to longitudinal displacement. These relationships remain conceptual and should not be interpreted as evidence that a particular coil orientation reduces stress, improves retention, or lowers migration risk. Instead, the framework identifies coil orientation as a complementary geometric variable that can be investigated alongside established material, surface, and structural design strategies. Further finite element analysis, computational fluid dynamics, fluid–structure interaction, experimental, and clinical studies are required to determine whether orientation‐dependent biomechanical responses produce measurable differences in hydrodynamic performance, stent stability, and patient‐relevant outcomes.

## Funding

This research is funded by the Indonesian Endowment Fund for Education (LPDP) on behalf of the Indonesian Ministry of Higher Education, Science and Technology and managed under the EQUITY Program (Contract Nos. 4299/B3/DT.03.08/2025 and 3029/PKS/lTS/2025).

## Conflicts of Interest

The authors declare no conflicts of interest.

## Data Availability

The data that support the findings of this study are available from the corresponding author upon reasonable request.
